# What would an ‘ideal’ glaucoma examination be like? - A conjoint analysis of patients’ and physicians’ preferences

**DOI:** 10.1007/s10792-021-01960-5

**Published:** 2021-07-26

**Authors:** Daniel R. Muth, Aljoscha S. Neubauer, Annemarie Klingenstein, Ulrich Schaller, Siegfried G. Priglinger, Christoph W. Hirneiß

**Affiliations:** Department of Ophthalmology, University Hospital, LMU Munich, Munich, Germany

**Keywords:** Glaucoma, Conjoint analysis, Diagnostic tools, Profiles, Quality of life, Survey

## Abstract

**Purpose:**

To structurally determine patients’ and physicians’ preferences for glaucoma diagnostic methods in order to improve glaucoma patient care and improve patient compliance with follow-up visits.

**Methods:**

Forty-one patients with glaucoma and 32 ophthalmologists were included in this cross-sectional study. Profiles representing glaucoma examinations were created using conjoint analysis (CA). The following factors of a glaucoma examination method were evaluated: (1) examination comfort, (2) examination frequency, (3) follow-up examination necessary in case of suspicious result, (4) cost for the patient, (5) travel time to examination site, (6) sensitivity and (7) specificity of the examination method.

**Results:**

Preferences were highest in both groups for examination sensitivity, followed by cost and specificity for the patient group. For the physician group, specificity was second most important, followed by cost. Least important was travel time for the patients and follow-up examinations for the physicians.

**Conclusions:**

Participants would rather pay more and travel longer to get a highly sensitive examination. This form of care is present in university eye hospitals. Consequently, it would be advisable to enhance capacities of these centers. Outpatient practices that offer glaucoma service should be fully equipped and should employ a glaucoma specialist.

**Supplementary Information:**

The online version contains supplementary material available at 10.1007/s10792-021-01960-5.

## Introduction

Market research tools found their application in healthcare starting in the 1970s [1–8]. They can help to structure the obtained data using established methods. Furthermore, they provide a method to keep the number of questions in a survey as low as possible in order to keep the participants willing to complete the questionnaire assuring good data quality [9]. The conductors of this study chose 7 key factors that define a glaucoma examination method. Those factors were prompted using a questionnaire. If the participant would be asked for every possible combination and expression level of these factors the number of questions would exceed the tolerable amount for the respondent. Therefore, the established market research tool conjoint analysis (CA) was used to determine the preferences of the participating patients and physicians. Knowing what factors the respondents lay emphasis on can help to design new, ‘ideal’ glaucoma examination methods that are more fitted to patients’ and physicians’ needs. The ultimate goal is to reduce the number of glaucoma patients that get lost to follow-up and go blind due to insufficient glaucoma diagnostic and treatment.

## Materials and methods

Approval was obtained from the institutional review board (IRB) of the university eye hospital of the Ludwig-Maximilians-University, Munich, Germany. This study adheres to the tenets of Helsinki.

### Participants

This cross-sectional study was conducted in a German university-affiliated glaucoma center: Glaucoma Unit of the Department of Ophthalmology, Ludwig-Maximilians-University, Munich, Germany.

The study was designed to consist of two groups, a patient and a physician group.

The patient group consisted of 41 patients. Inclusion criteria for the patient group were age ≥ 18 years and a clinical diagnosis of open-angle glaucoma (OAG) that was medically controlled or post-surgery and monitored by regular check-ups. Patients not meeting the inclusion criteria were excluded from the study. Prior ocular surgery was not an exclusion criterion. None of the patients that were selected based on the inclusion and exclusion criteria refused to answer the questionnaire (rejection rate).

The physician group consisted of 30 ophthalmologists and 2 medical students working at the eye hospital at that time. Inclusion criteria for the physician group were medical studies or a medical degree.

### Statistical analysis

The general principle of the statistical testing in this study is also referred to as “discrete choice experiment”. To set up the questionnaire so-called “profiles” (or “cards”) were used. Each profile describes different factors of one glaucoma diagnostic examination method. Each factor has different levels of expression. Consequently, every profile is a package consisting of more favorable and less favorable factor levels for the respondent. For example, a fictious profile 1 states that examination 1 is “very comfortable” and, at the same time, “very expensive”. Fictious profile 2 states that examination 2 is “very affordable” and, at the same time, “very uncomfortable”. The respondent is asked to give each profile a total preference (score). If the respondent in our example prefers profile 1 over profile 2, one can reversely conclude that for this respondent comfort is more important than cost.

In the same reverse manner, the average importance of each factor and the estimated preference (utility estimate) of each factor level is compiled (decompositional approach [10]). The average importance is the percentage how a factor contributes to the total preference of a profile. The utility estimate is the prediction of the preference of a factor level of a newly created, untested profile. The utility estimates add up to the total profile preference of this untested profile.

One established method for decompositional analysis is conjoint analysis (CA). Conjoint analysis is a statistical tool that has its origin in market research. It estimates what features of a new product will be most important to the customer (relative importance) [4–7, 11–18]. Therefore, conjoint analysis helps to design a new product full of features that convince customers to buy the product.

### Development of the profiles

When testing with profiles, the number of questions can grow too large for the respondent. Another strength of CA is the automatic reduction of the number of necessary profiles to a statistical representative minimum. This is achieved by combining the single factors and their levels.

In our study, each profile represented one glaucoma examination method with the following seven factors: (1) examination comfort, (2) frequency how often the examination needs to be performed, (3) follow-up examination necessary in case of suspicious result in order to confirm result, (4) cost for the patient, (5) travel time to examination site, (6) sensitivity and (7) specificity of the examination method.

The following expression levels for each factor were defined to build the profiles:

- *Comfort* (1) Comfortable and quick; (2) slightly uncomfortable and a few minutes; (3) very uncomfortable and 15 min (**3 levels**)
- *Frequency* (1) once; (2) every five years; (3) every two years; (4) every year (**4 levels**)
- *Follow-up* (1) no; (2) yes, (**2 levels**)
- *Cost* (1) none; (2) 10€; (3) 20€; (4) 70€; (5) 140€ (**5 levels**)
- *Travel time* (1) < 30 min.; (2) ca. 1 h; (3) ca. 2 h (**3 levels**)
- *Sensitivity* (1) 40%; (2) 70%; (3) 90% (**3 levels**)
- *Specificity* (1) 50%; (2) 80%; (3) 90% (**3 levels**)

### Conjoint analysis

The above-mentioned levels allow to generate 3*4*2*5*3*3*3 = 3240 profiles (full profile design) [11]. As 3240 would have been to many for the survey, the number of profiles presented to the respondents was reduced to a statistically representative subset (fractioned factorial design). This subset of profiles is called orthogonal array [11]. This was done using SPSS statistics (IBM Corporation). The function in SPSS is called “orthoplan”. The minimum number of profiles needed was automatically determined by the orthoplan function in SPSS. For this study, a number of 32 profiles was calculated. Four additional “holdout” profiles were created for internal validation, adding up to a total number of 36 profiles. The holdout profiles were ordered randomly with the other profiles and were given to the respondents to be answered. The respondents did not know which of the profiles were the holdouts. The results of the holdout profiles were not included in the main analysis by SPSS. They were only used for comparing the assessed results with the predicted results by the conjoint analysis.

In this study, the score version of the conjoint analysis was used, meaning that the participants had to assign a whole numbered score to each profile. The assessed scores were ordinal data. The derived utility values were metric.

To facilitate the statistical conjoint analysis, the assigned school grades were internally switched to “5” being “very good” and “1” being “insufficient”.

As SPSS does not include an already pre-compiled function for conjoint analysis, a SPSS syntax had to be written to generate the profiles and to perform the conjoint analysis. The SPSS syntax is shown in **Electronic Supplementary Material (ESM) 1**.

**Fig. 1:**
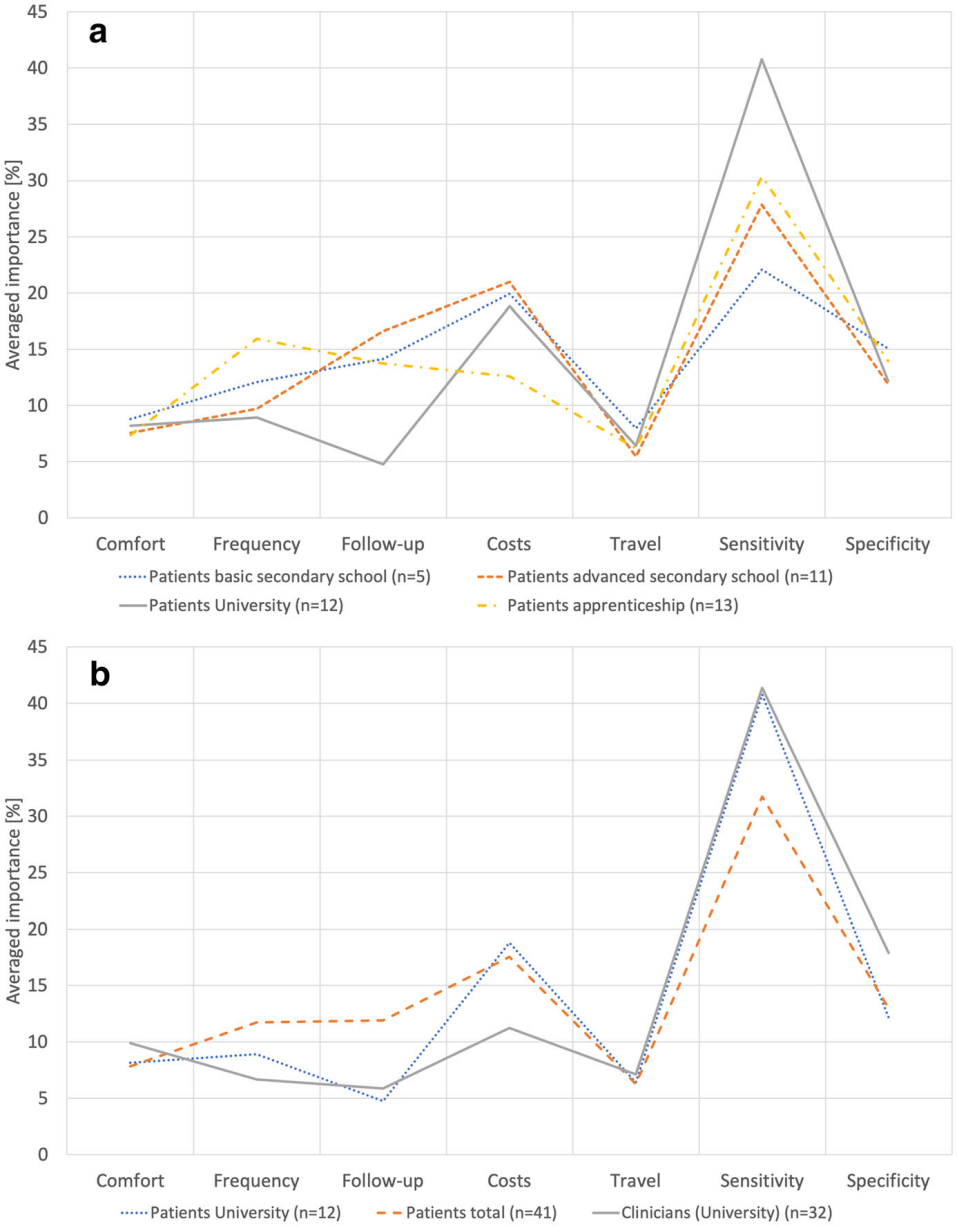
**a** Comparison of importance according to level of education. **b** Comparison of importance according to level of education

**Fig. 2:**
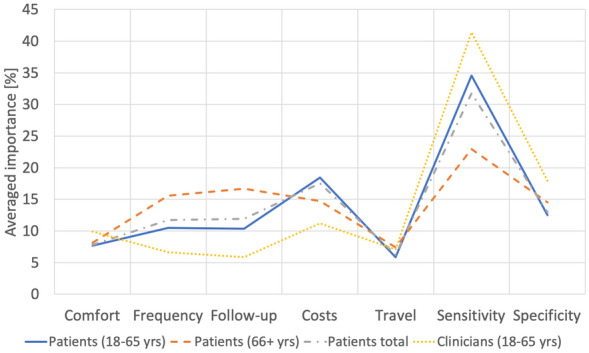
Comparison of importance according to age group

- The analysis was done for both groups as a whole as well as for sub-groups of the patients. One criterion to sub-group the patients was highest level of education (see Fig. 1a, b). Another sub-group division was made according to the patients’ age representing two sociodemographic phases of life: (1) “education and work” (18–65 years) and (2) “retirement” (≥ 66 years) (see Fig. 2).

The conjoint analysis procedure correlates the assessed preferences (via questionnaire) with the predicted preferences (for untested new profiles) and calculates a Pearson’s R and a Kendall’s tau (*τ*) correlation. A statistically significant and highly correlating coefficient indicates that the predictability of preferences is good.

Internal validation of the conjoint analysis is based on the same principle. Assessed preferences of the holdout cards are automatically correlated with the predicted preferences of the holdout cards by the conjoint analysis function. A high correlation indicates that the prediction algorithm matches the respondents’ preference [19].

For each factor within the conjoint syntax, it is deposited if a higher (“linear more”) or a lower (“linear less”) level is objectively considered more favorable. For example, cost is labeled “linear less” as less cost at same quality is regarded better. If a respondent gives a preference that is the opposite of the deposited level expression this is called a “reversal”. A high number of reversals can indicate that the respondents did not correctly understand the questions they answered.

Furthermore, conjoint analysis produces a so-called “B coefficient”. The B coefficient multiplied with level number of a factor calculates the utility estimate of the factor level (e.g., Frequency: “(**2**) Every 5 years”: **2** • − 0.007813 = − 0.0015625).

### Descriptive statistical analyses

Descriptive statistical analyses were performed to characterize the patients’ clinical, functional, and structural data.

The type 1 error, alpha (*α*), was defined as *α* = 0.05, implying that the level of statistical significance was at 0.05. Therefore, the probability value (*p*) had to be equal to or smaller than 0.05 to assume statistical significance (*p* ≤ 0.05). The software SPSS (version 22 for MacOS, IBM Corp.) was used to perform the statistical analysis.

### Conduct of the interviews

All profiles were administered as print-outs in 12 pt Arial font using Microsoft Word (Microsoft Office for Windows; Microsoft Corporation, Redmond, WA, USA). An example of a translated profile is shown in **Electronic Supplementary Material (ESM) 2**. The complete German questionnaire is available as electronic supplementary material (**ESM 3)**.

In the patient group, the questionnaires with the 36 profiles were personally handed to eligible patients who presented for scheduled follow-ups in the glaucoma department by one examiner (CWH). Informed consent was obtained of each voluntary participant. Before completing the survey, each participant was explained the meaning of the different factors and their levels to ensure sufficient understanding of the factors to be evaluated. Furthermore, the following sociodemographic data were recorded: (1) age, (2) sex, (3) highest level of education, (4) occupation (Table 1). The participants were then asked to rate each of the 36 profiles with a whole numbered score ranging from “1—Very good” to “5—Insufficient”. All participants should be most familiar with this kind of rating scale as it is similarly used at school.

**Table 1:** Characteristics of patient group and physician control group

	Patient group	Physician group
No. of participants (*n*)	41	32
Age [y] arithmetic mean (min.–max.)	54 (22–78)	32 (25–61)
(1) 18–65	31 (76%)	32 (100%)
(3) ≥ 66	10 (24%)	
Sex ratio (male %)	Male 39%	Male 34%
Level of education (after 4 years primary school)	5 (12%)	0
(1) Lower secondary education (5–6 years)	11 (27%)	0
(2) Advanced secondary school (6–9 years)	12 (29%)	32 (100%)
(3) University (3–6 years)	13 (32%)	0
(4) Apprenticeship (3 years)		
Current occupation		
(1) Full-time job	14 (34%)	28 (88%)
(2) Part-time job	6 (15%)	2 (6%)
(3) Student	1 (2%)	2 (6%)
(4) Retired	17 (42%)	0
(5) Disabled	2 (5%)	0
(6) Unemployed	0	0
(7) Other	1 (2%)	0
Experience in glaucoma diagnostic techniques	4 (10%)	12 (38%)
(1) None	17 (42%)	4 (13%)
(2) 1 examination technique (e.g., VF)	20 (48%)	15 (47%)
(3) > 1 examination technique	0	1 (3%)
Missing entries		

*y* years; *sec.* secondary; *VF* visual field

## Results

### Participants

Detailed demographic data are listed in Table 1.

### Preferences

The utility estimates for all factor levels in the two groups are displayed in Table 2. Higher utility represents higher preference, negative values lower preference.

**Table 2:** Utility estimates for each factor level

		Patient group		Physician group	
Factor	Factor level	Utility	SE	Utility	SE
Comfort	(1) Not uncomfortable, very fast	− 0.120011	0.051544	− 0.171165	0.048961
	(2) Slightly uncomfortable, few min	− 0.240022	0.103088	− 0.342330	0.097921
	(3) Very uncomfortable, 15 min	− 0.360033	0.154632	− 0.513494	0.146882
Frequency	(1) Examination only once	− 0.055793	0.038226	− 0.007813	0.036310
	(2) Examination every 5 years	− 0.111585	0.076452	− 0.015625	0.072620
	(3) Examination every 2 years	− 0.167378	0.114678	− 0.023438	0.108931
	(4) Examination every year	− 0.223171	0.152904	− 0.031250	0.145241
Follow-up needed	(1) No further follow-up	0.285061	0.085476	0.042969	0.081192
	(2) Yes, further follow-ups	0.570122	0.170952	0.085938	0.162384
Cost	(1) No cost, 0€	− 0.130466	0.032452	− 0.114161	0.030826
	(2) 10€ per examination	− 0.260932	0.064904	− 0.228322	0.061651
	(3) 20€ per examination	− 0.391397	0.097356	− 0.342483	0.092477
	(4) 70€ per examination	− 0.521863	0.129809	− 0.456644	0.123303
	(5) 140€ per examination	− 0.652329	0.162261	− 0.570805	0.154128
Travel time	(1) Less than 30 min	− 0.032428	0.051544	− 0.090199	0.048961
	(2) Ca. 60 min	− 0.064856	0.103088	− 0.180398	0.097921
	(3) Ca. 120 min	− 0.097284	0.154632	− 0.270597	0.146882
Sensitivity	(1) 40%	0.537417	0.051544	0.855824	0.048961
	(2) 70%	1.074834	0.103088	1.711648	0.097921
	(3) 90%	1.612251	0.154632	2.567472	0.146882
Specificity	(1) 50%	0.167129	0.051544	0.368608	0.048961
	(2) 80%	0.334257	0.103088	0.737216	0.097921
	(3) 90%	0.501386	0.154632	1.105824	0.146882

*Utility* utility estimate; *SE* standard error; *min.* minutes

Patients and physicians alike preferred comfortable examinations that can be performed quickly and only need to be done once. Furthermore, the examinations should be included in the basic health care insurance and therefore be free of charge. In case of a suspicious finding, both groups preferred follow-up examinations after the initial examination. The travel time to the examination site was desired to be low for both groups. Both groups also agreed on a simultaneously high statistical sensitivity and a high specificity.

**Fig. 3:**
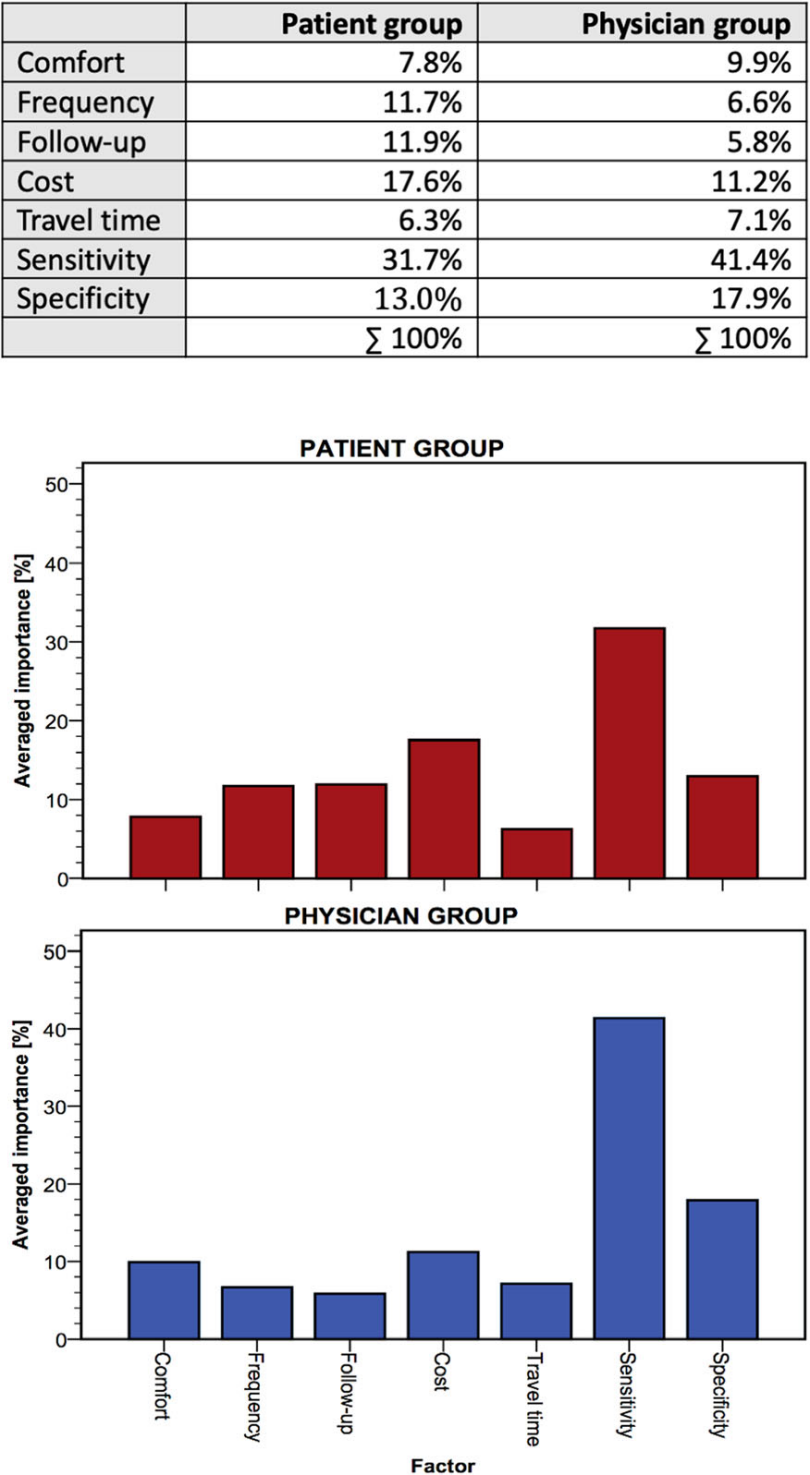
Average importance values [%] for each factor

The total average importance values in percent are shown in Fig. 3. They express for each group which factor had the greatest influence on the total preference of a profile, regardless the factor level. The single importance values add up to 100% per group.

The patient group as well as the physician group showed highest preferences for the sensitivity of the examination method (32% and 41%, respectively), followed by specificity (18%) and cost (11%) for the physician group. The patient group, in contrast, rated cost (18%) higher than specificity (13%). Comfort ranked fourth for patients and physicians with 8% and 10%, respectively. Follow-up examinations were the fifth important factor for the patient group (12%), whereas this was travel time for the physicians (7%). The examination frequency ranked sixth for both groups (12% and 7%, respectively). Least important for the patients was the travel time (6%) and the follow-up examinations for the physicians (6%).

With higher level of education, the importance of the examination sensitivity increased and the importance cost decreased (Fig. 1a, b). Stratified by age, the importance of cost also decreased with higher age (Fig. 2). Travel time became more important with increasing age. The importance of test specificity increased with age, whereas sensitivity reduced.

Both groups presented with Pearson's and Kendall's correlation coefficients greater than 0.9 and 0.7, respectively. The holdout profiles of the physician group showed a good internal validity with a high correlation between predicted and assessed preference values (*τ* = 1.000; *p* = 0.021). The patient’s group holdout was not statistically significant (*τ* = 0.667; *P* = 0.087) implying that patients often preferred more unfavorable options. The same image can be drawn by the number reversals. A reversal indicates that the participant’s choice was the opposite as expected, e.g., a participant preferred higher cost over lower cost (regardless the quality). The reversals in this study are listed in Table 3. The generated B coefficients and correlation values are displayed in Table 3.

**Table 3:** B coefficients and correlations

Patient group				Physician group		
		B coefficients	No. of reversals		B coefficients	No. of reversals
Comfort		− 0.120011	7		− 0.171165	6
Frequency		− 0.055793	15		− 0.007813	16
Follow-up		0.285061	27		0.042969	16
Cost		− 0.130466	9		− 0.114161	2
Travel time		− 0.032428	17		− 0.090199	9
Sensitivity		0.537417	6		0.855824	0
Specificity		0.167129	11		0.368608	1
		No. of subjects	No. of reversals		No. of subjects	No. of reversals
		6	1		15	1
		20	2		13	2
		8	3		3	3
		3	4			
		2	5			
	Correlation	*p*		Correlation	*p*	
Pearson’s R	0.930329	< 0.001		0.970717	< 0.001	
Kendall’s tau	0.732800	< 0.001		0.886647	< 0.001	
Kendall’s tau for 4 holdout profile cards	0.666667	0.087116		1.000000	0.020770	

*p* probability value

## Discussion

In terms of sensitivity, preferences of patients and of medical staff complied. Both groups preferred a test that securely uncovers diseased persons or persons with progressed illness. In contrast to the physician group, the patient group laid much importance on cost, more than on specificity. This is remarkable as all German citizens are covered by a basic health care insurance. Calculating a sub-group analysis within the patient group stratified by literacy (Fig. 1a, b) revealed that with higher level of education, sensitivity became more important than cost. This might imply that higher educated people tend to better understand the relevant factors of a medical examination method. Furthermore, it is to assume that higher educated and older people do have a better financial income, making cost less important. For the patient group, the importance of cost decreased with increasing age. Eye health seems to be valued more with progressed age. Young people do not seem to care that much about travel time and about possible follow-up examinations as those factors were rated less important in this age group.

While the calculated utility values for each factor level did not show any reversal trend, there were a considerable number of reversals found within the survey results. The high number of reversals for the factor “follow-up” could mean that the participants did not trust a single examination method and preferred a follow-up (with a different examination technique). Furthermore, the reversals for follow-up might also be explained by an ambiguous phrasing of the survey question. The intention was to say that the initial examination was not sufficient and a follow-up examination was necessary in case of suspicious result in order to confirm that result. The high number of reversals for the factor “frequency” indicates that patients with a chronic progressive disease such as glaucoma rather prefer to be monitored more closely although the examination method allows longer intervals.

It was striking that the holdout profiles of the patient group did not show a statistically significant correlation, whereas the other profiles correlated very well and significantly. Most probably this was due to the small number of participants in this group. *Aspinall *et al. suggest that the sample size (*n*) for a conjoint analysis should be at minimum (500·*L*)/(*F*·*S*) [20] with *L* being the maximum number of levels per factor, *F* being the total number of factors and *S* being the number of score values that can be made by the participant. According to this formula, *n* = (500·5)/(7·5) ≈ 72 participants would have been needed per group. When performing the sub-group analysis, *n* was smaller. This must be kept in mind when interpreting the results. It might have been easier to recruit more participants and to reduce the number of incomplete surveys if the number of profiles was lower and (some of) the questions less complex. Ideally, the patient was able to complete the questionnaire during his waiting time in the hospital.

Furthermore, it has to be taken into account that the results of this study are based on the German healthcare system. Although the study site belongs to one of Europe’s largest eye hospitals, the main ethnicity of patients is Caucasian and covered by the German public healthcare system. Therefore, the findings of this study might have limited generalizability to other populations with different demographics and healthcare systems. Health insurance status is likely to influence importance values [21]. Furthermore, our questionnaire did not assess the disease severity of the glaucoma patients participating in this study. Progress of disease might alter the importance values of the respondents [22]. Moreover, the study took place in a university hospital setting. Consequently, the participating medical staff is used to the diagnostic abilities that are easily available at a university with ophthalmological full supply. It must be borne in mind that the answers of the medical staff might differ among colleagues working in an office setting.

In summary, this study showed that all participants preferred a highly sensitive glaucoma examination. Looking at existing examination methods, there are techniques that do meet some of the preferences. For example, OCT and cSLO like the Heidelberg Retina Tomograph are highly sensitive, comfortable and fast. As these diagnostic tools are expensive they can only be offered by university eye hospitals and larger ophthalmic centers. Consequently, it would be advisable to enhance capacities of these centers. Outpatient practices that offer glaucoma service should be fully equipped and should employ a glaucoma specialist. If more of these centers existed in rural areas, travel time could significantly be decreased and compliance with follow-ups improved.

## Supplementary Information

Below is the link to the electronic supplementary material.

Supplementary file1 (DOCX 17 KB)

Supplementary file2 (PDF 129 KB)

Supplementary file3 (PDF 305 KB)
