# Supplementary material for: What would an ‘ideal’ glaucoma examination be like? - A conjoint analysis of patients’ and physicians’ preferences
Source: Int Ophthalmol. 2021 Jul 26;41(12):3911–20. doi: 10.1007/s10792-021-01960-5 (PMC8572838; doi:10.1007/s10792-021-01960-5)
Supplement: Supplementary file 1 — Supplementary file1 (DOCX 17 KB) [file 10792_2021_1960_MOESM1_ESM.docx]

**Supplemental Digital Content (SDC 1).** SPSS syntax for generating profile cards

**Generate Orthogonal Design.*

*SET SEED 123.*

*ORTHOPLAN*

*/FACTORS=Comfort 'Examination comfort' (1 'Not uncomfortable, very fast' 2 'Uncomfortable, few minutes' 3 'Very uncomfortable, 15 minutes')*

*Frequency 'Examination frequency' (1 'Once' 2 'Every 5 years' 3 'Every 2 years' 4 'Every year')*

*Followup 'Follow-ups necessary' (1 'No follow-ups' 2 'Yes, follow-ups needed')*

*Cost 'Cost per examination' (1 'No cost' 2 '10 Euros' 3 '20 Euros' 4 '70 Euros' 5 '140 Euros')*

*Travel 'Travel time to examination' (1 'Less than 30 minutes' 2 'Ca. 60 minutes' 3 'Ca. 120 minutes')*

*Sens 'Sensitivity' (1 '40%' 2 '70%' 3 '90%')*

*Spec 'Specificity' (1 '50%' 2 '80%' 3 '90%')*

*/OUTFILE='[output location of file with profiles].sav'*

*/HOLDOUT 4*

*/MIXHOLD YES.*

SPSS-Syntax for performing conjoint analysis:

*CONJOINT*

*PLAN=[import location for plan file with profiles].SAV''*

*/DATA='[import location of data].SAV'*

*/SCORE=profile1 TO profile36*

*/SUBJECT=ID*

*/FACTORS=Comfort (LINEAR LESS) Frequency (LINEAR LESS) Followup (LINEAR LESS) Cost (LINEAR LESS) Travel (LINEAR LESS) Sens (LINEAR MORE) Spec (LINEAR MORE)*

*/UTILITY='[output location of file with calculated utility values].SAV'*

*/PLOT=ALL*

*/PRINT=ALL.*
