# Supplementary material for: What would an ‘ideal’ glaucoma examination be like? - A conjoint analysis of patients’ and physicians’ preferences
Source: Int Ophthalmol. 2021 Jul 26;41(12):3911–20. doi: 10.1007/s10792-021-01960-5 (PMC8572838; doi:10.1007/s10792-021-01960-5)
Supplement: Supplementary file 2 — Supplementary file2 (PDF 129 KB) [file 10792_2021_1960_MOESM2_ESM.pdf]

University Eye Hospital of the
Ludwig-Maximilians-University Munich

Ludwig-Maximilians-Universität München

Director: Prof. Dr. med. Siegfried Priglinger

Klinikum der Universität München • Augenklinik – Innenstadt
Mathildenstraße 8 • D-80336 München

Telephone (089) 4400 53811 (Reception)
Telefax (089) 4400- (Reception)

Prof. Dr. med Christoph W. Hirneiss , Daniel R. Muth

Translated version Feb. 26, 2017

Tel.: +49 89 4400 - 53811

Glaucoma diagnostics – Patients' preferences

Dear patient,

in order to fit the used glaucoma examination techniques better to your preferences we are conducting a survey. First, we would like to ask you to give some anonymized data about yourself. Then, we would like you to evaluate 36 **completely theoretical** examination techniques. Each technique is presented with characteristic features. You are asked **to give a school grade to each of the techniques ranging from "1 – (very good)" to "5 – (insufficient)":**

1	2	3	4	5
very good				insufficient

Please base your rating of the examination techniques on how acceptable such a examination technique would be for you. Thank you very much!

Annotation:

All examination techniques are completely theoretical and do not influence the techniques currently used for you during your visit.

Confidentiality of the data:

Completely anonymized surveys only are used and no personal data is assessed. Therefore, it is not possible to trace any survey back to the person who completed it. The regulations of medical confidentiality and data protection are adhered. As the assessment as well as the analysis of the data is done completely anonymized transfer of personal data is not possible.

Vorlage KUM/OC – fe (00-03-13)

Your sex

☐ Female ☐ Male

Your age ……………………………..years

What is you highest degree of education?

☐ Secondary modern school (German “Hauptschule“)

☐ Other secondary school (junior high (“Realschule“), grammar school (”Gymnasium”), cooperative education (“Berufsakademie”, etc.)

☐ University studies

☐ Apprenticeship

☐ Other ________________________________

What is your current occupation?

☐ Full-time job

☐ Part-time job

☐ Student

☐ Retired

☐ Unemployable

☐ Unemployed

☐ Other ________________________________

How experienced are you in glaucoma diagnostics (examination techniques)?

☐ I have not experienced any glaucoma examination technique so far

☐ I have already experienced one glaucoma examination technique so far (e.g. visual field testing, Heidelberg Retina Tomography (HRT))

☐ I have already experienced several (more than one) glaucoma examination techniques so far

**Examination technique number**	1
**Examination technique**	Not uncomfortable, very fast
**Examination frequency**	Examination every year
**Follow-up examination needed in case of suspicious results**	Yes, follow-ups are needed in case of suspicious results
**Costs**	You have to pay 70,- € per examination
**Travel time to location where examination is performed**	Less than 30 minutes
**When 10 persons WITH glaucoma or WITH glaucoma progression are examined with this technique...**	… 9 persons (90%) are identified as ill or worse correctly by this examination technique (sensitivity) ■■■■■■■■■□
**When 10 persons WITHOUT glaucoma or WITHOUT progression of their glaucoma disease are examined with this technique...**	... 9 persons (90%) are identified as eye healthy or with stable disease correctly (specifity) ■■■■■■■■■□

Your rating of this examination technique:

1	2	3	4	5
very good				insufficient
