# Supplementary material for: What would an ‘ideal’ glaucoma examination be like? - A conjoint analysis of patients’ and physicians’ preferences
Source: Int Ophthalmol. 2021 Jul 26;41(12):3911–20. doi: 10.1007/s10792-021-01960-5 (PMC8572838; doi:10.1007/s10792-021-01960-5)
Supplement: Supplementary file 3 — Supplementary file3 (PDF 305 KB) [file 10792_2021_1960_MOESM3_ESM.pdf]

Klinikum der Universität München
Augenklinik – Innenstadt
Direktor: Prof. Dr. med. Anselm Kampik

Ludwig-Maximilians-Universität München

Klinikum der Universität München • Augenklinik – Innenstadt
Mathildenstraße 8 • D-80336 München

Telefon (089) 5160-3811 (Pforte)
Telefax (089) 5160-5160 (Pforte)

Dr. C. Hirneiss

Version 27.09.2009

Tel.: (089) 5160 - 3811

Glaukomdiagnostik - Patientenpräferenzen

Sehr geehrte Patientin, sehr geehrter Patient,

um die eingesetzten Untersuchungsverfahren bei Glaukom noch besser auf Ihre Wünsche abstimmen zu können, führen wir eine Befragung durch. Hierzu möchten wir Sie bitten, einige anonymisierte persönliche Angaben zu machen und dann 36, **rein theoretische** Verfahren zu beurteilen. Jedes Verfahren wird mit einigen Eigenschaften dargestellt. Sie sollen dann jeweils das **Verfahren insgesamt mit Schulnoten von 1 (sehr gut) bis 5 (mangelhaft) bewerten**:

1	2	3	4	5
sehr gut				mangelhaft

Gehen Sie bei Ihrer Bewertung davon aus, wie akzeptabel für Sie persönlich eine Untersuchung mit einem solchen Verfahren wäre. Danke !

Anmerkung:

Alle Verfahren sind rein theoretisch konstruiert und haben keinen Einfluss darauf, welche Untersuchungen derzeit bei Ihnen eingesetzt werden.

Vertraulichkeit der Daten

Es werden nur komplett anonymisierte Bögen verwendet und keinerlei personenbezogene Daten erfasst. Es ist deshalb kein Rückschluss auf die die Befragung ausfüllende Person möglich. Die Bestimmungen der ärztlichen Schweigepflicht und des Datenschutzes sind gewährleistet. Da die Datenerhebung und Auswertung komplett anonym erfolgt, ist eine Weitergabe personenbezogener Daten ausgeschlossen.

KUM/OC – fe (00-03-13)

Ihr Geschlecht

☐ Weiblich ☐ Männlich

Ihr Alter …………………………….. Jahre

Was ist Ihr höchster Schulabschluss ?

☐ Hauptschule

☐ weiterführende Schulform (Realschule, Gymnasium, Berufsakademie, etc.)

☐ Studium

☐ Berufsausbildung

☐ Andere ________________________________

Sind Sie derzeit erwerbstätig ?

☐ Vollzeit erwerbstätig

☐ Teilzeit erwerbstätig

☐ Student

☐ **in Rente**

☐ erwerbsunfähig

☐ arbeitslos

☐ Andere ________________________________

Welche Erfahrung haben Sie bisher mit Glaukom-Diagnostik ?

☐ Kein Verfahren selbst erlebt

☐ Ein Verfahren selbst erlebt (z.B. Gesichtsfeld, HRT)

☐ Mehrere unterschiedliche Diagnostik-Verfahren selbst erlebt

Verfahren Nummer	1
**Untersuchungskomfort**	Nicht unangenehm, sehr schnell
**Häufigkeit der Untersuchung**	jährliche Untersuchung
**Bestätigung im Falle von Auffälligkeit**	Weitere Untersuchung bei Auffälligkeit
**Kosten für Sie**	Sie zahlen 70 € pro Untersuchung
**Reisezeit bis zum Untersuchungsort**	weniger als 30 Min zum Untersuchungsort
**Wenn 10 Personen MIT Glaukom -oder MIT Verschlechterung bei Glaukom-diesen Test erhalten, dann**	werden hiervon 9 Personen (90%) richtig als krank /verschlechtert erkannt<br>■■■■■■■■■□
**Wenn 10 Personen OHNE Glaukom -oder OHNE Verschlechterung bei Glaukom-diesen Test erhalten, dann**	werden hiervon 9 Personen (90%) richtig als unverändert erkannt<br>■■■■■■■■■□

Ihre Bewertung des Verfahrens:

1<br>sehr gut	2	3	4	5<br>mangelhaft

Verfahren Nummer	2
**Untersuchungskomfort**	Nicht unangenehm, sehr schnell
**Häufigkeit der Untersuchung**	jährliche Untersuchung
**Bestätigung im Falle von Auffälligkeit**	Keine weitere Untersuchung bei Auffälligkeiten nötig
**Kosten für Sie**	Sie zahlen 140 € pro Untersuchung
**Reisezeit bis zum Untersuchungsort**	etwa 120 Min (2 h) zum Untersuchungsort
**Wenn 10 Personen MIT Glaukom -oder MIT Verschlechterung bei Glaukom-diesen Test erhalten, dann**	werden hiervon 4 Personen (40%) richtig als krank /verschlechtert erkannt<br>■■■■□□□□□□
**Wenn 10 Personen OHNE Glaukom -oder OHNE Verschlechterung bei Glaukom-diesen Test erhalten, dann**	werden hiervon 8 Personen (80%) richtig als unverändert erkannt.<br>■■■■■■■■□□

Ihre Bewertung des Verfahrens:

1<br>sehr gut	2	3	4	5<br>mangelhaft

Verfahren Nummer	3
**Untersuchungskomfort**	etwas unangenehm&anstrengend, wenige Min
**Häufigkeit der Untersuchung**	Einmalige Untersuchung
**Bestätigung im Falle von Auffälligkeit**	Weitere Untersuchung bei Auffälligkeit
**Kosten für Sie**	Sie zahlen 140 € pro Untersuchung
**Reisezeit bis zum Untersuchungsort**	weniger als 30 Min zum Untersuchungsort
**Wenn 10 Personen MIT Glaukom -oder MIT Verschlechterung bei Glaukom-diesen Test erhalten, dann**	werden hiervon 9 Personen (90%) richtig als krank /verschlechtert erkannt<br>■■■■■■■■■□
**Wenn 10 Personen OHNE Glaukom -oder OHNE Verschlechterung bei Glaukom-diesen Test erhalten, dann**	werden hiervon 5 Personen (50%) richtig als unverändert erkannt.<br>■■■■■□□□□□

Ihre Bewertung des Verfahrens:

1<br>sehr gut	2	3	4	5<br>mangelhaft

Verfahren Nummer	4
**Untersuchungskomfort**	sehr anstrengend&ungangenehm, 15 min
**Häufigkeit der Untersuchung**	Einmalige Untersuchung
**Bestätigung im Falle von Auffälligkeit**	Keine weitere Untersuchung bei Auffälligkeiten nötig
**Kosten für Sie**	Sie zahlen 20 € pro Untersuchung
**Reisezeit bis zum Untersuchungsort**	weniger als 30 Min zum Untersuchungsort
**Wenn 10 Personen MIT Glaukom -oder MIT Verschlechterung bei Glaukom-diesen Test erhalten, dann**	werden hiervon 7 Personen (70%) richtig als krank /verschlechtert erkannt<br>■■■■■■■□□□
**Wenn 10 Personen OHNE Glaukom -oder OHNE Verschlechterung bei Glaukom-diesen Test erhalten, dann**	werden hiervon 9 Personen (90%) richtig als unverändert erkannt<br>■■■■■■■■■□

Ihre Bewertung des Verfahrens:

1<br>sehr gut	2	3	4	5<br>mangelhaft

Verfahren Nummer	5
**Untersuchungskomfort**	etwas unangenehm&anstrengend, wenige Min
**Häufigkeit der Untersuchung**	Untersuchung alle 2 Jahre
**Bestätigung im Falle von Auffälligkeit**	Weitere Untersuchung bei Auffälligkeit
**Kosten für Sie**	Sie zahlen 10 € pro Untersuchung
**Reisezeit bis zum Untersuchungsort**	etwa 120 Min (2 h) zum Untersuchungsort
**Wenn 10 Personen MIT Glaukom -oder MIT Verschlechterung bei Glaukom- diesen Test erhalten, dann**	werden hiervon 7 Personen (70%) richtig als krank /verschlechtert erkannt ■■■■■■■□□□
**Wenn 10 Personen OHNE Glaukom - oder OHNE Verschlechterung bei Glaukom-diesen Test erhalten, dann**	werden hiervon 5 Personen (50%) richtig als unverändert erkannt. ■■■■■□□□□□

Ihre Bewertung des Verfahrens:

1	2	3	4	5
sehr gut				mangelhaft

Verfahren Nummer	6
**Untersuchungskomfort**	etwas unangenehm&anstrengend, wenige Min
**Häufigkeit der Untersuchung**	jährliche Untersuchung
**Bestätigung im Falle von Auffälligkeit**	Weitere Untersuchung bei Auffälligkeit
**Kosten für Sie**	Keine Kosten
**Reisezeit bis zum Untersuchungsort**	etwa 60 Min (1 h) zum Untersuchungsort
**Wenn 10 Personen MIT Glaukom -oder MIT Verschlechterung bei Glaukom- diesen Test erhalten, dann**	werden hiervon 7 Personen (70%) richtig als krank /verschlechtert erkannt ■■■■■■■□□□
**Wenn 10 Personen OHNE Glaukom - oder OHNE Verschlechterung bei Glaukom-diesen Test erhalten, dann**	werden hiervon 8 Personen (80%) richtig als unverändert erkannt. ■■■■■■■■□□

Ihre Bewertung des Verfahrens

1	2	3	4	5
sehr gut				mangelhaft

**Verfahren Nummer**	7
**Untersuchungskomfort**	Nicht unangenehm, sehr schnell
**Häufigkeit der Untersuchung**	jährliche Untersuchung
**Bestätigung im Falle von Auffälligkeit**	Keine weitere Untersuchung bei Auffälligkeiten nötig
**Kosten für Sie**	Keine Kosten
**Reisezeit bis zum Untersuchungsort**	weniger als 30 Min zum Untersuchungsort
**Wenn 10 Personen MIT Glaukom -oder MIT Verschlechterung bei Glaukom-diesen Test erhalten, dann**	werden hiervon 7 Personen (70%) richtig als krank /verschlechtert erkannt ■■■■■■■□□□
**Wenn 10 Personen OHNE Glaukom -oder OHNE Verschlechterung bei Glaukom-diesen Test erhalten, dann**	werden hiervon 5 Personen (50%) richtig als unverändert erkannt. ■■■■■□□□□□

Ihre Bewertung des Verfahrens:

1	2	3	4	5
sehr gut				mangelhaft

**Verfahren Nummer**	8
**Untersuchungskomfort**	Nicht unangenehm, sehr schnell
**Häufigkeit der Untersuchung**	Einmalige Untersuchung
**Bestätigung im Falle von Auffälligkeit**	Weitere Untersuchung bei Auffälligkeit
**Kosten für Sie**	Sie zahlen 10 € pro Untersuchung
**Reisezeit bis zum Untersuchungsort**	weniger als 30 Min zum Untersuchungsort
**Wenn 10 Personen MIT Glaukom -oder MIT Verschlechterung bei Glaukom-diesen Test erhalten, dann**	werden hiervon 4 Personen (40%) richtig als krank /verschlechtert erkannt ■■■■□□□□□□
**Wenn 10 Personen OHNE Glaukom -oder OHNE Verschlechterung bei Glaukom-diesen Test erhalten, dann**	werden hiervon 9 Personen (90%) richtig als unverändert erkannt ■■■■■■■■■□

Ihre Bewertung des Verfahrens

1	2	3	4	5
sehr gut				mangelhaft

Verfahren Nummer	9
**Untersuchungskomfort**	sehr anstrengend&ungangenehm, 15 min
**Häufigkeit der Untersuchung**	Untersuchung alle 2 Jahre
**Bestätigung im Falle von Auffälligkeit**	Weitere Untersuchung bei Auffälligkeit
**Kosten für Sie**	Sie zahlen 20 € pro Untersuchung
**Reisezeit bis zum Untersuchungsort**	weniger als 30 Min zum Untersuchungsort
**Wenn 10 Personen MIT Glaukom -oder MIT Verschlechterung bei Glaukom-diesen Test erhalten, dann**	werden hiervon 4 Personen (40%) richtig als krank /verschlechtert erkannt<br>■■■■□□□□□□
**Wenn 10 Personen OHNE Glaukom -oder OHNE Verschlechterung bei Glaukom-diesen Test erhalten, dann**	werden hiervon 8 Personen (80%) richtig als unverändert erkannt.<br>■■■■■■■■□□

Ihre Bewertung des Verfahrens:

1	2	3	4	5
sehr gut				mangelhaft

Verfahren Nummer	10
**Untersuchungskomfort**	Nicht unangenehm, sehr schnell
**Häufigkeit der Untersuchung**	Untersuchung alle 2 Jahre
**Bestätigung im Falle von Auffälligkeit**	Weitere Untersuchung bei Auffälligkeit
**Kosten für Sie**	Sie zahlen 140 € pro Untersuchung
**Reisezeit bis zum Untersuchungsort**	etwa 60 Min (1 h) zum Untersuchungsort
**Wenn 10 Personen MIT Glaukom -oder MIT Verschlechterung bei Glaukom-diesen Test erhalten, dann**	werden hiervon 4 Personen (40%) richtig als krank /verschlechtert erkannt<br>■■■■□□□□□□
**Wenn 10 Personen OHNE Glaukom -oder OHNE Verschlechterung bei Glaukom-diesen Test erhalten, dann**	werden hiervon 9 Personen (90%) richtig als unverändert erkannt<br>■■■■■■■■■□

Ihre Bewertung des Verfahrens

1	2	3	4	5
sehr gut				mangelhaft

Verfahren Nummer	11
**Untersuchungskomfort**	sehr anstrengend&ungangenehm, 15 min
**Häufigkeit der Untersuchung**	Untersuchung alle 5 Jahre
**Bestätigung im Falle von Auffälligkeit**	Weitere Untersuchung bei Auffälligkeit
**Kosten für Sie**	Sie zahlen 70 € pro Untersuchung
**Reisezeit bis zum Untersuchungsort**	etwa 60 Min (1 h) zum Untersuchungsort
**Wenn 10 Personen MIT Glaukom -oder MIT Verschlechterung bei Glaukom-diesen Test erhalten, dann**	werden hiervon 4 Personen (40%) richtig als krank /verschlechtert erkannt<br>■■■■□□□□□□
**Wenn 10 Personen OHNE Glaukom -oder OHNE Verschlechterung bei Glaukom-diesen Test erhalten, dann**	werden hiervon 5 Personen (50%) richtig als unverändert erkannt.<br>■■■■■□□□□□

Ihre Bewertung des Verfahrens:

1<br>sehr gut	2	3	4	5<br>mangelhaft

Verfahren Nummer	12
**Untersuchungskomfort**	sehr anstrengend&ungangenehm, 15 min
**Häufigkeit der Untersuchung**	jährliche Untersuchung
**Bestätigung im Falle von Auffälligkeit**	Weitere Untersuchung bei Auffälligkeit
**Kosten für Sie**	Sie zahlen 10 € pro Untersuchung
**Reisezeit bis zum Untersuchungsort**	weniger als 30 Min zum Untersuchungsort
**Wenn 10 Personen MIT Glaukom -oder MIT Verschlechterung bei Glaukom-diesen Test erhalten, dann**	werden hiervon 4 Personen (40%) richtig als krank /verschlechtert erkannt<br>■■■■□□□□□□
**Wenn 10 Personen OHNE Glaukom -oder OHNE Verschlechterung bei Glaukom-diesen Test erhalten, dann**	werden hiervon 5 Personen (50%) richtig als unverändert erkannt.<br>■■■■■□□□□□

Ihre Bewertung des Verfahrens

1<br>sehr gut	2	3	4	5<br>mangelhaft

Verfahren Nummer	13
**Untersuchungskomfort**	sehr anstrengend&ungangenehm, 15 min
**Häufigkeit der Untersuchung**	Einmalige Untersuchung
**Bestätigung im Falle von Auffälligkeit**	Weitere Untersuchung bei Auffälligkeit
**Kosten für Sie**	Sie zahlen 70 € pro Untersuchung
**Reisezeit bis zum Untersuchungsort**	etwa 60 Min (1 h) zum Untersuchungsort
**Wenn 10 Personen MIT Glaukom -oder MIT Verschlechterung bei Glaukom-diesen Test erhalten, dann**	werden hiervon 7 Personen (70%) richtig als krank /verschlechtert erkannt ■■■■■■■□□□
**Wenn 10 Personen OHNE Glaukom -oder OHNE Verschlechterung bei Glaukom-diesen Test erhalten, dann**	werden hiervon 5 Personen (50%) richtig als unverändert erkannt. ■■■■■□□□□□

Ihre Bewertung des Verfahrens:

1 sehr gut	2	3	4	5 mangelhaft

Verfahren Nummer	14
**Untersuchungskomfort**	Nicht unangenehm, sehr schnell
**Häufigkeit der Untersuchung**	Einmalige Untersuchung
**Bestätigung im Falle von Auffälligkeit**	Weitere Untersuchung bei Auffälligkeit
**Kosten für Sie**	Sie zahlen 20 € pro Untersuchung
**Reisezeit bis zum Untersuchungsort**	etwa 60 Min (1 h) zum Untersuchungsort
**Wenn 10 Personen MIT Glaukom -oder MIT Verschlechterung bei Glaukom-diesen Test erhalten, dann**	werden hiervon 7 Personen (70%) richtig als krank /verschlechtert erkannt ■■■■■■■□□□
**Wenn 10 Personen OHNE Glaukom -oder OHNE Verschlechterung bei Glaukom-diesen Test erhalten, dann**	werden hiervon 5 Personen (50%) richtig als unverändert erkannt. ■■■■■□□□□□

Ihre Bewertung des Verfahrens

1 sehr gut	2	3	4	5 mangelhaft

Verfahren Nummer	15
**Untersuchungskomfort**	Nicht unangenehm, sehr schnell
**Häufigkeit der Untersuchung**	Untersuchung alle 2 Jahre
**Bestätigung im Falle von Auffälligkeit**	Weitere Untersuchung bei Auffälligkeit
**Kosten für Sie**	Keine Kosten
**Reisezeit bis zum Untersuchungsort**	weniger als 30 Min zum Untersuchungsort
**Wenn 10 Personen MIT Glaukom -oder MIT Verschlechterung bei Glaukom- diesen Test erhalten, dann**	werden hiervon 9 Personen (90%) richtig als krank /verschlechtert erkannt ■■■■■■■■■□
**Wenn 10 Personen OHNE Glaukom - oder OHNE Verschlechterung bei Glaukom-diesen Test erhalten, dann**	werden hiervon 5 Personen (50%) richtig als unverändert erkannt. ■■■■■□□□□□

Ihre Bewertung des Verfahrens:

1	2	3	4	5
sehr gut				mangelhaft

Verfahren Nummer	16
**Untersuchungskomfort**	sehr anstrengend&ungangenehm, 15 min
**Häufigkeit der Untersuchung**	jährliche Untersuchung
**Bestätigung im Falle von Auffälligkeit**	Keine weitere Untersuchung bei Auffälligkeiten nötig
**Kosten für Sie**	Sie zahlen 10 € pro Untersuchung
**Reisezeit bis zum Untersuchungsort**	etwa 60 Min (1 h) zum Untersuchungsort
**Wenn 10 Personen MIT Glaukom -oder MIT Verschlechterung bei Glaukom- diesen Test erhalten, dann**	werden hiervon 9 Personen (90%) richtig als krank /verschlechtert erkannt ■■■■■■■■■□
**Wenn 10 Personen OHNE Glaukom - oder OHNE Verschlechterung bei Glaukom-diesen Test erhalten, dann**	werden hiervon 5 Personen (50%) richtig als unverändert erkannt. ■■■■■□□□□□

Ihre Bewertung des Verfahrens

1	2	3	4	5
sehr gut				mangelhaft

Verfahren Nummer	17
**Untersuchungskomfort**	etwas unangenehm&anstrengend, wenige Min
**Häufigkeit der Untersuchung**	Einmalige Untersuchung
**Bestätigung im Falle von Auffälligkeit**	Keine weitere Untersuchung bei Auffälligkeiten nötig
**Kosten für Sie**	Sie zahlen 70 € pro Untersuchung
**Reisezeit bis zum Untersuchungsort**	etwa 120 Min (2 h) zum Untersuchungsort
**Wenn 10 Personen MIT Glaukom -oder MIT Verschlechterung bei Glaukom-diesen Test erhalten, dann**	werden hiervon 4 Personen (40%) richtig als krank /verschlechtert erkannt ■■■■□□□□□□
**Wenn 10 Personen OHNE Glaukom -oder OHNE Verschlechterung bei Glaukom-diesen Test erhalten, dann**	werden hiervon 5 Personen (50%) richtig als unverändert erkannt. ■■■■■□□□□□

Ihre Bewertung des Verfahrens:

1 sehr gut	2	3	4	5 mangelhaft

Verfahren Nummer	18
**Untersuchungskomfort**	Nicht unangenehm, sehr schnell
**Häufigkeit der Untersuchung**	jährliche Untersuchung
**Bestätigung im Falle von Auffälligkeit**	Weitere Untersuchung bei Auffälligkeit
**Kosten für Sie**	Sie zahlen 20 € pro Untersuchung
**Reisezeit bis zum Untersuchungsort**	etwa 120 Min (2 h) zum Untersuchungsort
**Wenn 10 Personen MIT Glaukom -oder MIT Verschlechterung bei Glaukom-diesen Test erhalten, dann**	werden hiervon 4 Personen (40%) richtig als krank /verschlechtert erkannt ■■■■□□□□□□
**Wenn 10 Personen OHNE Glaukom -oder OHNE Verschlechterung bei Glaukom-diesen Test erhalten, dann**	werden hiervon 5 Personen (50%) richtig als unverändert erkannt. ■■■■■□□□□□

Ihre Bewertung des Verfahrens

1 sehr gut	2	3	4	5 mangelhaft

Verfahren Nummer	19
**Untersuchungskomfort**	Nicht unangenehm, sehr schnell
**Häufigkeit der Untersuchung**	Untersuchung alle 5 Jahre
**Bestätigung im Falle von Auffälligkeit**	Keine weitere Untersuchung bei Auffälligkeiten nötig
**Kosten für Sie**	Sie zahlen 20 € pro Untersuchung
**Reisezeit bis zum Untersuchungsort**	etwa 120 Min (2 h) zum Untersuchungsort
**Wenn 10 Personen MIT Glaukom -oder MIT Verschlechterung bei Glaukom-diesen Test erhalten, dann**	werden hiervon 9 Personen (90%) richtig als krank /verschlechtert erkannt<br>■■■■■■■■■□
**Wenn 10 Personen OHNE Glaukom -oder OHNE Verschlechterung bei Glaukom-diesen Test erhalten, dann**	werden hiervon 5 Personen (50%) richtig als unverändert erkannt.<br>■■■■■□□□□□

Ihre Bewertung des Verfahrens:

1	2	3	4	5
sehr gut				mangelhaft

Verfahren Nummer	20
**Untersuchungskomfort**	etwas unangenehm&anstrengend, wenige Min
**Häufigkeit der Untersuchung**	jährliche Untersuchung
**Bestätigung im Falle von Auffälligkeit**	Keine weitere Untersuchung bei Auffälligkeiten nötig
**Kosten für Sie**	Sie zahlen 20 € pro Untersuchung
**Reisezeit bis zum Untersuchungsort**	weniger als 30 Min zum Untersuchungsort
**Wenn 10 Personen MIT Glaukom -oder MIT Verschlechterung bei Glaukom-diesen Test erhalten, dann**	werden hiervon 4 Personen (40%) richtig als krank /verschlechtert erkannt<br>■■■■□□□□□□
**Wenn 10 Personen OHNE Glaukom -oder OHNE Verschlechterung bei Glaukom-diesen Test erhalten, dann**	werden hiervon 9 Personen (90%) richtig als unverändert erkannt<br>■■■■■■■■■□

Ihre Bewertung des Verfahrens

1	2	3	4	5
sehr gut				mangelhaft

Verfahren Nummer	21
**Untersuchungskomfort**	etwas unangenehm&anstrengend, wenige Min
**Häufigkeit der Untersuchung**	jährliche Untersuchung
**Bestätigung im Falle von Auffälligkeit**	Weitere Untersuchung bei Auffälligkeit
**Kosten für Sie**	Sie zahlen 20 € pro Untersuchung
**Reisezeit bis zum Untersuchungsort**	weniger als 30 Min zum Untersuchungsort
**Wenn 10 Personen MIT Glaukom -oder MIT Verschlechterung bei Glaukom- diesen Test erhalten, dann**	werden hiervon 4 Personen (40%) richtig als krank /verschlechtert erkannt ■■■■□□□□□□
**Wenn 10 Personen OHNE Glaukom - oder OHNE Verschlechterung bei Glaukom-diesen Test erhalten, dann**	werden hiervon 8 Personen (80%) richtig als unverändert erkannt. ■■■■■■■■□□

Ihre Bewertung des Verfahrens:

1	2	3	4	5
sehr gut				mangelhaft

Verfahren Nummer	22
**Untersuchungskomfort**	Nicht unangenehm, sehr schnell
**Häufigkeit der Untersuchung**	Untersuchung alle 5 Jahre
**Bestätigung im Falle von Auffälligkeit**	Keine weitere Untersuchung bei Auffälligkeiten nötig
**Kosten für Sie**	Sie zahlen 10 € pro Untersuchung
**Reisezeit bis zum Untersuchungsort**	weniger als 30 Min zum Untersuchungsort
**Wenn 10 Personen MIT Glaukom -oder MIT Verschlechterung bei Glaukom- diesen Test erhalten, dann**	werden hiervon 4 Personen (40%) richtig als krank /verschlechtert erkannt ■■■■□□□□□□
**Wenn 10 Personen OHNE Glaukom - oder OHNE Verschlechterung bei Glaukom-diesen Test erhalten, dann**	werden hiervon 8 Personen (80%) richtig als unverändert erkannt. ■■■■■■■■□□

Ihre Bewertung des Verfahrens

1	2	3	4	5
sehr gut				mangelhaft

Verfahren Nummer	23
**Untersuchungskomfort**	Nicht unangenehm, sehr schnell
**Häufigkeit der Untersuchung**	Einmalige Untersuchung
**Bestätigung im Falle von Auffälligkeit**	Keine weitere Untersuchung bei Auffälligkeiten nötig
**Kosten für Sie**	Keine Kosten
**Reisezeit bis zum Untersuchungsort**	weniger als 30 Min zum Untersuchungsort
**Wenn 10 Personen MIT Glaukom -oder MIT Verschlechterung bei Glaukom-diesen Test erhalten, dann**	werden hiervon 4 Personen (40%) richtig als krank /verschlechtert erkannt ■■■■□□□□□□
**Wenn 10 Personen OHNE Glaukom -oder OHNE Verschlechterung bei Glaukom-diesen Test erhalten, dann**	werden hiervon 5 Personen (50%) richtig als unverändert erkannt. ■■■■■□□□□□

Ihre Bewertung des Verfahrens:

1 sehr gut	2	3	4	5 mangelhaft

Verfahren Nummer	24
**Untersuchungskomfort**	Nicht unangenehm, sehr schnell
**Häufigkeit der Untersuchung**	Untersuchung alle 2 Jahre
**Bestätigung im Falle von Auffälligkeit**	Keine weitere Untersuchung bei Auffälligkeiten nötig
**Kosten für Sie**	Sie zahlen 20 € pro Untersuchung
**Reisezeit bis zum Untersuchungsort**	etwa 60 Min (1 h) zum Untersuchungsort
**Wenn 10 Personen MIT Glaukom -oder MIT Verschlechterung bei Glaukom-diesen Test erhalten, dann**	werden hiervon 4 Personen (40%) richtig als krank /verschlechtert erkannt ■■■■□□□□□□
**Wenn 10 Personen OHNE Glaukom -oder OHNE Verschlechterung bei Glaukom-diesen Test erhalten, dann**	werden hiervon 5 Personen (50%) richtig als unverändert erkannt. ■■■■■□□□□□

Ihre Bewertung des Verfahrens

1 sehr gut	2	3	4	5 mangelhaft

**Verfahren Nummer**	25
**Untersuchungskomfort**	sehr anstrengend&ungangenehm, 15 min
**Häufigkeit der Untersuchung**	Untersuchung alle 2 Jahre
**Bestätigung im Falle von Auffälligkeit**	Keine weitere Untersuchung bei Auffälligkeiten nötig
**Kosten für Sie**	Keine Kosten
**Reisezeit bis zum Untersuchungsort**	etwa 120 Min (2 h) zum Untersuchungsort
**Wenn 10 Personen MIT Glaukom -oder MIT Verschlechterung bei Glaukom-diesen Test erhalten, dann**	werden hiervon 9 Personen (90%) richtig als krank /verschlechtert erkannt
**Wenn 10 Personen OHNE Glaukom -oder OHNE Verschlechterung bei Glaukom-diesen Test erhalten, dann**	werden hiervon 9 Personen (90%) richtig als unverändert erkannt ■■■■■■■■■□

Ihre Bewertung des Verfahrens:

1	2	3	4	5
sehr gut				mangelhaft

**Verfahren Nummer**	26
**Untersuchungskomfort**	Nicht unangenehm, sehr schnell
**Häufigkeit der Untersuchung**	Untersuchung alle 5 Jahre
**Bestätigung im Falle von Auffälligkeit**	Weitere Untersuchung bei Auffälligkeit
**Kosten für Sie**	Keine Kosten
**Reisezeit bis zum Untersuchungsort**	weniger als 30 Min zum Untersuchungsort
**Wenn 10 Personen MIT Glaukom -oder MIT Verschlechterung bei Glaukom-diesen Test erhalten, dann**	werden hiervon 4 Personen (40%) richtig als krank /verschlechtert erkannt ■■■■□□□□□□
**Wenn 10 Personen OHNE Glaukom -oder OHNE Verschlechterung bei Glaukom-diesen Test erhalten, dann**	werden hiervon 5 Personen (50%) richtig als unverändert erkannt. ■■■■■□□□□□

Ihre Bewertung des Verfahrens

1	2	3	4	5
sehr gut				mangelhaft

Verfahren Nummer	27
**Untersuchungskomfort**	Nicht unangenehm, sehr schnell
**Häufigkeit der Untersuchung**	Untersuchung alle 5 Jahre
**Bestätigung im Falle von Auffälligkeit**	Weitere Untersuchung bei Auffälligkeit
**Kosten für Sie**	Sie zahlen 10 € pro Untersuchung
**Reisezeit bis zum Untersuchungsort**	etwa 120 Min (2 h) zum Untersuchungsort
**Wenn 10 Personen MIT Glaukom -oder MIT Verschlechterung bei Glaukom-diesen Test erhalten, dann**	werden hiervon 7 Personen (70%) richtig als krank /verschlechtert erkannt<br>■■■■■■■□□□
**Wenn 10 Personen OHNE Glaukom -oder OHNE Verschlechterung bei Glaukom-diesen Test erhalten, dann**	werden hiervon 9 Personen (90%) richtig als unverändert erkannt<br>■■■■■■■■■□

Ihre Bewertung des Verfahrens:

1<br>sehr gut	2	3	4	5<br>mangelhaft

Verfahren Nummer	28
**Untersuchungskomfort**	Nicht unangenehm, sehr schnell
**Häufigkeit der Untersuchung**	Untersuchung alle 2 Jahre
**Bestätigung im Falle von Auffälligkeit**	Weitere Untersuchung bei Auffälligkeit
**Kosten für Sie**	Sie zahlen 10 € pro Untersuchung
**Reisezeit bis zum Untersuchungsort**	etwa 60 Min (1 h) zum Untersuchungsort
**Wenn 10 Personen MIT Glaukom -oder MIT Verschlechterung bei Glaukom-diesen Test erhalten, dann**	werden hiervon 9 Personen (90%) richtig als krank /verschlechtert erkannt<br>■■■■■■■■■□
**Wenn 10 Personen OHNE Glaukom -oder OHNE Verschlechterung bei Glaukom-diesen Test erhalten, dann**	werden hiervon 9 Personen (90%) richtig als unverändert erkannt<br>■■■■■■■■■□

Ihre Bewertung des Verfahrens

1<br>sehr gut	2	3	4	5<br>mangelhaft

Verfahren Nummer	29
**Untersuchungskomfort**	Nicht unangenehm, sehr schnell
**Häufigkeit der Untersuchung**	Einmalige Untersuchung
**Bestätigung im Falle von Auffälligkeit**	Keine weitere Untersuchung bei Auffälligkeiten nötig
**Kosten für Sie**	Sie zahlen 10 € pro Untersuchung
**Reisezeit bis zum Untersuchungsort**	etwa 60 Min (1 h) zum Untersuchungsort
**Wenn 10 Personen MIT Glaukom -oder MIT Verschlechterung bei Glaukom-diesen Test erhalten, dann**	werden hiervon 9 Personen (90%) richtig als krank /verschlechtert erkannt ■■■■■■■■■□
**Wenn 10 Personen OHNE Glaukom -oder OHNE Verschlechterung bei Glaukom-diesen Test erhalten, dann**	werden hiervon 8 Personen (80%) richtig als unverändert erkannt. ■■■■■■■■□□

**Ihre Bewertung des Verfahrens:**	**1** **sehr gut**	**2**	**3**	**4**	**5** **mangelhaft**

Verfahren Nummer	30
**Untersuchungskomfort**	etwas unangenehm&anstrengend, wenige Min
**Häufigkeit der Untersuchung**	Untersuchung alle 2 Jahre
**Bestätigung im Falle von Auffälligkeit**	Keine weitere Untersuchung bei Auffälligkeiten nötig
**Kosten für Sie**	Sie zahlen 10 € pro Untersuchung
**Reisezeit bis zum Untersuchungsort**	weniger als 30 Min zum Untersuchungsort
**Wenn 10 Personen MIT Glaukom -oder MIT Verschlechterung bei Glaukom-diesen Test erhalten, dann**	werden hiervon 4 Personen (40%) richtig als krank /verschlechtert erkannt ■■■■□□□□□□
**Wenn 10 Personen OHNE Glaukom -oder OHNE Verschlechterung bei Glaukom-diesen Test erhalten, dann**	werden hiervon 5 Personen (50%) richtig als unverändert erkannt. ■■■■■□□□□□

**Ihre Bewertung des Verfahrens**	**1** **sehr gut**	**2**	**3**	**4**	**5** **mangelhaft**

Verfahren Nummer	31
**Untersuchungskomfort**	etwas unangenehm&anstrengend, wenige Min
**Häufigkeit der Untersuchung**	Untersuchung alle 5 Jahre
**Bestätigung im Falle von Auffälligkeit**	Keine weitere Untersuchung bei Auffälligkeiten nötig
**Kosten für Sie**	Keine Kosten
**Reisezeit bis zum Untersuchungsort**	etwa 60 Min (1 h) zum Untersuchungsort
**Wenn 10 Personen MIT Glaukom -oder MIT Verschlechterung bei Glaukom-diesen Test erhalten, dann**	werden hiervon 4 Personen (40%) richtig als krank /verschlechtert erkannt<br>■■■■□□□□□□
**Wenn 10 Personen OHNE Glaukom -oder OHNE Verschlechterung bei Glaukom-diesen Test erhalten, dann**	werden hiervon 9 Personen (90%) richtig als unverändert erkannt<br>■■■■■■■■■□

Ihre Bewertung des Verfahrens:

1<br>sehr gut	2	3	4	5<br>mangelhaft

Verfahren Nummer	32
**Untersuchungskomfort**	sehr anstrengend&ungangenehm, 15 min
**Häufigkeit der Untersuchung**	Einmalige Untersuchung
**Bestätigung im Falle von Auffälligkeit**	Weitere Untersuchung bei Auffälligkeit
**Kosten für Sie**	Keine Kosten
**Reisezeit bis zum Untersuchungsort**	etwa 120 Min (2 h) zum Untersuchungsort
**Wenn 10 Personen MIT Glaukom -oder MIT Verschlechterung bei Glaukom-diesen Test erhalten, dann**	werden hiervon 4 Personen (40%) richtig als krank /verschlechtert erkannt<br>■■■■□□□□□□
**Wenn 10 Personen OHNE Glaukom -oder OHNE Verschlechterung bei Glaukom-diesen Test erhalten, dann**	werden hiervon 8 Personen (80%) richtig als unverändert erkannt.<br>■■■■■■■■□□

Ihre Bewertung des Verfahrens

1<br>sehr gut	2	3	4	5<br>mangelhaft

Verfahren Nummer	33
**Untersuchungskomfort**	sehr anstrengend&ungangenehm, 15 min
**Häufigkeit der Untersuchung**	jährliche Untersuchung
**Bestätigung im Falle von Auffälligkeit**	Keine weitere Untersuchung bei Auffälligkeiten nötig
**Kosten für Sie**	Sie zahlen 70 € pro Untersuchung
**Reisezeit bis zum Untersuchungsort**	etwa 120 Min (2 h) zum Untersuchungsort
**Wenn 10 Personen MIT Glaukom -oder MIT Verschlechterung bei Glaukom- diesen Test erhalten, dann**	werden hiervon 9 Personen (90%) richtig als krank /verschlechtert erkannt ■■■■■■■■■□
**Wenn 10 Personen OHNE Glaukom - oder OHNE Verschlechterung bei Glaukom-diesen Test erhalten, dann**	werden hiervon 9 Personen (90%) richtig als unverändert erkannt ■■■■■■■■■□

Ihre Bewertung des Verfahrens:

1	2	3	4	5
sehr gut				mangelhaft

Verfahren Nummer	34
**Untersuchungskomfort**	etwas unangenehm&anstrengend, wenige Min
**Häufigkeit der Untersuchung**	Untersuchung alle 5 Jahre
**Bestätigung im Falle von Auffälligkeit**	Weitere Untersuchung bei Auffälligkeit
**Kosten für Sie**	Sie zahlen 20 € pro Untersuchung
**Reisezeit bis zum Untersuchungsort**	weniger als 30 Min zum Untersuchungsort
**Wenn 10 Personen MIT Glaukom -oder MIT Verschlechterung bei Glaukom- diesen Test erhalten, dann**	werden hiervon 9 Personen (90%) richtig als krank /verschlechtert erkannt ■■■■■■■■■□
**Wenn 10 Personen OHNE Glaukom - oder OHNE Verschlechterung bei Glaukom-diesen Test erhalten, dann**	werden hiervon 8 Personen (80%) richtig als unverändert erkannt. ■■■■■■■■□□

Ihre Bewertung des Verfahrens

1	2	3	4	5
sehr gut				mangelhaft

Verfahren Nummer	35
**Untersuchungskomfort**	sehr anstrengend&ungangenehm, 15 min
**Häufigkeit der Untersuchung**	Untersuchung alle 5 Jahre
**Bestätigung im Falle von Auffälligkeit**	Keine weitere Untersuchung bei Auffälligkeiten nötig
**Kosten für Sie**	Sie zahlen 140 € pro Untersuchung
**Reisezeit bis zum Untersuchungsort**	weniger als 30 Min zum Untersuchungsort
**Wenn 10 Personen MIT Glaukom -oder MIT Verschlechterung bei Glaukom-diesen Test erhalten, dann**	werden hiervon 7 Personen (70%) richtig als krank /verschlechtert erkannt ■■■■■■■□□□
**Wenn 10 Personen OHNE Glaukom -oder OHNE Verschlechterung bei Glaukom-diesen Test erhalten, dann**	werden hiervon 5 Personen (50%) richtig als unverändert erkannt. ■■■■■□□□□□

Ihre Bewertung des Verfahrens:

1 sehr gut	2	3	4	5 mangelhaft

Verfahren Nummer	36
**Untersuchungskomfort**	Nicht unangenehm, sehr schnell
**Häufigkeit der Untersuchung**	Untersuchung alle 2 Jahre
**Bestätigung im Falle von Auffälligkeit**	Keine weitere Untersuchung bei Auffälligkeiten nötig
**Kosten für Sie**	Sie zahlen 70 € pro Untersuchung
**Reisezeit bis zum Untersuchungsort**	weniger als 30 Min zum Untersuchungsort
**Wenn 10 Personen MIT Glaukom -oder MIT Verschlechterung bei Glaukom-diesen Test erhalten, dann**	werden hiervon 7 Personen (70%) richtig als krank /verschlechtert erkannt ■■■■■■■□□□
**Wenn 10 Personen OHNE Glaukom -oder OHNE Verschlechterung bei Glaukom-diesen Test erhalten, dann**	werden hiervon 8 Personen (80%) richtig als unverändert erkannt. ■■■■■■■■□□

Ihre Bewertung des Verfahrens

1 sehr gut	2	3	4	5 mangelhaft
